# Editorial: Fatigability and Motor Performance in Special and Clinical Populations

**DOI:** 10.3389/fphys.2020.570861

**Published:** 2021-02-11

**Authors:** Inge Zijdewind, Allison Hyngstrom, Sandra Hunter

**Affiliations:** ^1^Biomedical Sciences of Cells and Systems, University Medical Center Groningen, University of Groningen, Groningen, Netherlands; ^2^Department of Physical Therapy, Marquette University, Milwaukee, WI, United States

**Keywords:** fatigue, cognition, motor performance, elderly, women, clinical populations

Clinical and special populations often report higher levels of fatigue than typically presenting age-matched persons. Despite the high incidence of fatigue, the responsible mechanisms are still poorly understood. This is, partly, a consequence of confusing terminology. Enoka et al. (Kluger et al., 2013; Enoka and Duchateau, 2016), therefore, provided a conceptual framework that defined fatigue as a disabling symptom with attributes of performance fatigability and perceived fatigability. Performance fatigability is defined as the decline in an objective measure of performance (typically motor performance) over time. Perceived fatigability reflects the sensations which accompany regulation of the integrity of the performer and depends on the psychological state and the physiological capacity of the body to maintain homeostasis. Perceived fatigability is measured as a self-reported rating and can be evaluated as a state or trait both during motor performance or at rest.

Performance and perceived fatigability are both relevant to clinical populations and can limit physical and cognitive function. Typically, performance and perceived fatigability are studied as separate attributes in healthy and clinical populations. Consequently, there is minimal understanding of how these attributes of fatigue integrate to affect motor performance and well-being, particularly in clinical populations.

Past research has typically concentrated on the mechanisms that limit performance fatigability during single limb isometric or dynamic contractions, often in healthy young males, with little distinction between males and females, or consideration of race or ethnicity. In populations such as females, old adults, and clinical populations (e.g., people with multiple sclerosis, stroke, cancer, obesity, diabetes, amyotrophic lateral sclerosis, Parkinson's disease, and spinal cord injury to name a few), there is limited knowledge on performance fatigability, the involved mechanisms that limit performance, how fatigability assessed in the laboratory predicts functional tasks in the real world, and the interactions with perceived fatigability. This Research Topic highlights several studies and reviews on: (1) the debilitating effects of perceived fatigability in several clinical populations, and associations with performance fatigability, and (2), the mechanisms of performance fatigability, and the effects on function in various populations, including older adults, females after child birth, stroke survivors, people who are obese, and those with mild traumatic brain injury (MTBI), chronic respiratory disease (CRD) rheumatic arthritis heart failure and multiple sclerosis (MS). Below we highlight the common themes and what can be learned from these collective studies.

## Perceived and Performance Fatigability in Clinical and Special Populations

Many clinical populations report greater levels of perceived fatigability than age-matched controls [multiple sclerosis: (Gould et al.) mild traumatic brain injury: (Prak et al.) and also as highlighted in mini-reviews about people with rheumatoid arthritis (Marrelli et al.) chronic respiratory diseases (Gruet)]. However, despite the prevalence of perceived fatigability in some clinical populations, only a few manuscripts addressed the effects of perceived fatigability on performance fatigability or functional outcomes (Gruet). The mini-reviews emphasize the underreporting in the literature related to fatigue in people individuals with heart failure (Keller-Ross et al.), rheumatoid arthritis (Marrelli et al.), and chronic respiratory disease (Gruet). Thus, there is a need for studies on the interaction of perceptions of fatigue experienced in clinical populations, performance fatigability, and functional tasks.

Studies addressing whether performance fatigability differs between clinical populations and age-matched controls is more numerous than perceived fatigability, although the outcome appears to be dependent on the characteristics of the population, the involved muscle group, the requirements of the fatiguing task and the variable used to quantify fatigability. Several reviews provide examples of greater performance fatigability in populations with heart failure (Keller-Ross et al.) and rheumatoid arthritis (Marrelli et al.) than controls. In contrast, several original data studies report that performance fatigability is similar between clinical populations and controls including stroke survivors (Murphy et al.), and people with MTBI: (Prak et al.); and MS: (Gould et al.). The decline in isometric voluntary force of a single muscle groups (either the knee extensor muscles or finger abductors) was between 25 and 40% but not different between the clinical and control group. Similarly, other studies highlight in this Research Topic found no difference between other populations including young and older adults for a small muscle of the hand (Sars et al.) and between the knee extensor muscles of obese and non-obese older participants (Duan et al.). It is too simplistic however, to conclude that there is no difference in performance fatigability between a select clinical population and controls, or between young and old adults because fatigability is task dependent. The aging literature for example, shows that older adults are less fatigable than young adults for upper and lower limb muscles during an isometric-contraction fatiguing task but more fatigable when performing fast velocity contractions (Hunter et al., 2016). Similarly, based on contraction type, individuals with stroke were more fatigable than age-matched controls (Hyngstrom et al., 2012). Clearly, more studies are needed to determine performance fatigability with different task requirements that are functionally relevant.

To understand the mechanisms of performance fatigability, several studies obtained additional measurements to discriminate between a loss of force due to reductions in voluntary activation (neural drive) to the muscle and that due to altered contractile function (Gandevia, 2001). Variables known to reflect differences in voluntary activation are often more depressed in clinical and special populations whereas measures of contractile properties can be less affected for isometric fatiguing tasks (Murphy et al.; Sars et al.). Similar measurements in persons with MTBI (Prak et al.) however, only showed minor differences in voluntary drive and contractile function compared with controls. It is possible that while performance fatigability may not differ between groups, the mechanisms for failure of force during a fatiguing task can differ between populations and provide insight for strategies to address potential functional deficits and testing in different clinical populations. The mini-review by Gruet for example, presents several arguments to evaluate tests used to quantify performance fatigability in clinical populations.

## Fatigability and Functional Outcomes

One goal of studying perceived and performance fatigability, and muscle function in general is to determine how it affects functional performance in vulnerable populations in real-life situations. For example, older adults who performed prolonged walking as a fatiguing exercise exhibited greater declines and greater variability in the minimal toe clearance during walking than young adults (Watanabe), possibly exposing them to greater risk of falling. Furthermore, for fatiguing exercise of upper limb muscles, lower force steadiness was associated with (1) intellectual capacity in people with MS (Gould et al.), and (2) executive function in older adults who performed a dual motor and cognitive task (Pereira et al.). The mechanistic link between cognitive function and motor tasks with fatiguing exercise in these populations deserves greater exploration.

Lastly, fatiguing trunk flexor exercise lessened localized sensitivity to pain and decreased pain perception in postpartum women indicating that trunk exercises may be useful for acute pain relief for clinical populations that are characterized by pain and/or weakness in the abdominal region muscles (Deering et al.). Thus, fatigability maybe an important and under-quantified marker of overall function in clinical and special populations.

## Conclusion

The collective publications in this Research Topic, highlight the need and tremendous opportunity for high impact studies addressing fatigue and its relevance to functional performance in a variety of clinical and special populations. Such studies will provide a foundation for determining optimal rehabilitation strategies involving training/exercise protocols, drugs or other novel interventions to treat fatigue in clinical populations and whether there are differences between the sexes and with aging.

## Author Contributions

IZ, AH, and SH contributed to writing this editorial. All authors contributed to the article and approved the submitted version.

## Conflict of Interest

The authors declare that the research was conducted in the absence of any commercial or financial relationships that could be construed as a potential conflict of interest.
